# Interdependent Self-Construal Moderates Relationships Between Positive Emotion and Quality in Social Interactions: A Case of Person to Culture Fit

**DOI:** 10.3389/fpsyg.2019.00914

**Published:** 2019-04-30

**Authors:** Konstantinos G. Kafetsios

**Affiliations:** Department of Psychology, University of Crete, Rethymno, Greece

**Keywords:** culture, self-construal, emotion, social interaction, fit

## Abstract

How emotion is experienced and expressed in social encounters can very much depend on a person’s cultural orientation and the two can affect the quality of social relationships. The present research examined how an interaction between cultural orientation (person) and emotion in social encounters (situation) can influence social interaction outcomes and by extent, cultural fit. For a period of seven days, participants (*N* = 164) reported eight positive and eight negative emotions they experienced in naturally occurring social encounters together with indicators of quality of social interaction (satisfaction, attending to the other, perceiving others as emotionally more positive). Results from multilevel random coefficient analyses found that self-construal, interdependence in particular, moderated relationships between positive emotion and social interaction quality. At high levels of positive emotion, higher, compared to lower, interdependence was associated with lower attention to other and lower satisfaction with the encounter. At low levels of positive emotion, higher interdependence was associated with higher social interaction quality than persons lower in interdependence. These effects were more robust when social anxiety was controlled, and social anxiety was highly correlated with participants’ interdependent orientation. The results support socially situated accounts to emotion and cultural constructions of the self, and depict emotion in social interaction as an important indicator of cultural fit.

## Introduction

The emotions people experience and express in their day to day encounters are important for their well-being and life-satisfaction. This is especially the case with regard to positive emotion: sharing positive feelings with social acquaintances, friends, and partners can critically shape well-being (Gable et al., 2004; Lambert et al., 2013). Yet, not all persons evaluate positive emotion as psychologically beneficial and not all social contexts afford beneficial outcomes for positive emotion (Greenaway and Kalokerinos, 2017).

One key factor that determines the social costs and benefits of positive emotion is culture-related norms (Eid and Diener, 2001) and scripts^1^ (Miyamoto and Ma, 2011). Culture-related norms and scripts regarding how relationships are enacted can regulate the emotional experience in people’s social exchanges (Kim et al., 2008). In particular, individuals’ fit to their culture’s emotion norms and scripts can affect their psychological well-being (Nezlek et al., 2008; De Leersnyder et al., 2014). The present study meaningfully extends this research by looking at how emotion, especially positive emotion, can shape the quality of social interaction as a function of people’s self-construal and culture’s central cultural mandate.

Emotions are inherently social and as such they are influenced by culture-contingent modes of processing affective reactions (Keltner and Haidt, 1999). Affiliation and social distancing, two key social functions of emotion, are also culture-contingent (Fischer and Manstead, 2008). For example, in individualistic cultures where people tend to understand and define themselves as independent of others, socially disengaging emotions such as anger and contempt are more desirable. Conversely, in collectivistic cultures, where people tend to define themselves more in terms of relationships with others (Markus and Kitayama, 1991), socially engaging emotions such as sadness or guilt, are more desirable. The affiliative and social distancing functions of positive and negative emotion can thus shape cognitions and behaviors during social interactions (Pervin, 1976; Forgas, 2001).

Cultural differences are also evident with regard to the dominant norms and scripts for regulating positive and negative emotion in a given culture. Individualistic values and independent self-construal are associated with more intense and more frequent positive emotion, whereas interdependent views of the self are typically associated with less positive emotion (Van Hemert et al., 2007). Persons in individualistic cultures typically pursue and up-regulate positive emotion (Miyamoto and Ma, 2011). Norms of suppressing emotion which are prevalent in more collectivistic cultures (Matsumoto et al., 2008), on the other hand, have been held responsible for stronger negative and weaker positive emotions in those cultures and in social interactions within them (Kafetsios and Nezlek, 2012). Critically, as a result of a key overarching norm to preserve harmony within as well as between groups, in collectivistic cultures positive emotion is considered a threat to the harmony rule either within in-groups or in relationship to out-groups (Matsumoto, 1990) and this can critically affect how people relate emotionally with one another in their social encounters.

When culture and individual level self-construal converge, the above observations become even more pronounced. In an individualistic culture (United Kingdom), persons who were higher on independent self-construal, reported more positive emotion in their day to day social encounters than persons who were lower on independent self-construal; in a collectivistic culture (Greece), conversely, participants reporting higher independence experienced less positive emotion in their day to day encounters, compared to persons with lower independent self-construal (Nezlek et al., 2008). This evidence highlights a person to culture fit in terms of emotional aspects of social interactions. One’s cultural orientation and his/her fit within their culture’s central mandate can thus shape experience of emotion and its expression (Mesquita et al., 2016). It also illustrates that cultural orientations, independence and interdependence in particular, are not stable across cultures but can depend on the specific structuring situations that give rise to independent or interdependent mind sets (e.g., Hong et al., 2000; Oyserman and Sorensen, 2009) resulting in person by situation variations.

Notably, beyond a few studies, emotional fit to culture has not been explored in social interactions. This is despite recent evidence for a strong link between emotional fit with host culture and *relational* aspects of well-being (De Leersnyder et al., 2014; Jasini et al., 2018) and calls for the need to look at emotion as embedded within people’s social interactions (Fischer and Van Kleef, 2010). Important for the argument behind the present study is that situational factors can determine how cultural scripts are expressed in social behavior (Leu et al., 2010). Social interaction contexts, which are usually dyadic, may further intensify and prime culture-related scripts especially for those persons with an interdependent understanding of the self (Kafetsios and Hess, 2013), a cultural orientation that is particularly geared toward relationships with others (Hong et al., 2000).

Research recently has provided some evidence on how emotion in dyadic encounters can influence relational outcomes as a function of individuals’ chronic self-construal and overarching cultural scripts. In collectivistic cultures and for persons higher in chronic interdependence, negative emotion and emotion suppression had some positive outcomes in personal relationships (Randall et al., 2011; Le and Impett, 2013). A recent study that directly examined the role of affective tone in day to day social encounters in relation to interactants’ chronic self-construal (Kafetsios et al., 2018), found that persons higher in chronic interdependence, had less drop in satisfaction, acceptance and understanding perceptions in social interactions as a result of a drop in negative affect compared to persons lower in interdependence. This effect was observed only in the collectivistic culture (Greece); in the individualistic culture, Germany, positive emotion was predictive of higher quality social interaction for more independent persons. These results were explained with reference to the function of negative emotion in social encounters in collectivistic cultures and for persons higher in interdependent orientation. Cultural scripts where higher suppression and emotional closeness co-occur, can lead to negative emotions more weakly related to negative relational outcomes in social encounters for persons who hold those scripts more prominently (those higher on interdependence).

However, the role of positive emotion in social encounters in relation to persons’ cultural orientation, interdependence in particular, has not been adequately addressed. In Kafetsios et al. (2018) study, positive and negative affect valence was assessed with a single item. Hence, there was limited opportunity to examine variability in positive and negative emotion in people’s day to day encounters. Such a generic measure of emotion may have resulted in less nuanced emotion reports (Robinson and Clore, 2002), and reports which are closer to abstract constructions of emotion in the two cultures (Tsai, 2007).

Moreover, there are other individual-level factors related to self-construal that can interact with cultural norms and scripts to influence social interactions. One such is social anxiety. Social anxiety is a personality variable associated with general positivity and negativity in social encounters (Diener et al., 1999; Vassilopoulos and Banerjee, 2010) and is closely related to cultural constructions of the self. Persons with an interdependent self-construal, East Asians in particular, demonstrate increased levels of social anxiety (e.g., Okazaki, 1997; Lee et al., 2006). The utilization of non-verbal skills in social interaction has been identified as a mediator of social anxiety for highly interdependent persons (Lau et al., 2009). There has been limited research on social anxiety in actual social interactions and in non-Asian collectivistic cultures.

## The Present Study

The present study aimed to examine how chronic interdependent and independent self-construal within a collectivistic culture (Greece; Hofstede, 2001), can shape quality of social interaction as a function of proximal aspects of those interactions, especially positive emotion. Emotion carries significant relational information for social interactants (Fischer and Manstead, 2008), positive emotion in particular (Berry and Hansen, 1996), and persons with higher or lower independent and interdependent self-construal differ in how they utilize positive and negative emotion in their social relationships (Nezlek et al., 2008; Kafetsios et al., 2018).

The study examined a number of emotions participants experienced during their social interactions and how those emotions may interact with participants’ self-construal to predict the quality of social encounters. In particular, it was expected that due to culture-level harmony-preserving norms and scripts in a collectivistic culture, and higher prominence of those scripts for persons with an interdependent orientation, the positive relationship between positive emotion and quality in social interactions will be weakened (*H1*). Equally, the anticipated negative relationship between negative emotion and social interaction quality will be weakened as a function of higher interdependence, in line with previous research (Kafetsios et al., 2018, *H2*).

In order to ascertain that the observed relationships concern the affective valence of social relationships and not individual-level factors, person-level positive or negative affect was controlled. Social anxiety was also taken into consideration given its relationship with interdependent self-construal, yet there were no specific hypotheses regarding social anxiety effects in quality of social interaction.

## Materials and Methods

### Participants

There were 165 participants (37 men and 128 women) with a mean age of 24.31 years (*SD* = 6.05). In order to estimate the sample size, recommendations regarding sample sizes at individual and social interaction levels for random coefficient models were followed (Nezlek, 2011). Additionally, we estimated power based on an ordinary least square (OLS) power analysis calculator (G^∗^Power, Faul et al., 2007), according to which power for a medium effect (*f*^2^ = 0.15) with four predictors, *N* = 129, and alpha = 0.05 would be 0.95. Moreover, Table 1 presents the proportion of variance within persons as indicative of power for detecting within level and cross-level interaction effects (Mathieu et al., 2012).

**Table 1:** Zero order correlations and descriptive statistics of study main variables.

	1	2	3	4	5	6
(1) Gender	1					
(2) Independent	-0.11	1				
(3) Interdependent	0.12	-0.08	1			
(4) Social Anxiety	0.07	-0.31^**^	0.41^**^	1		
(5) Positive Affect	-0.09	0.11	0.05	0.08	1	
(6) Negative Affect	0.04	-0.04	0.05	0.06	-0.24^**^	1
*M*		4.74	4.51	2.14	2.97	1.73
(*SD*)		(0.58)	(0.68)	(0.95)	(0.60)	(0.50)


*^∗∗^*p* < 0.01.*

The data set included students recruited through posters and calls in lectures in a Greek state University and members of the community (30%). Following completion of the questionnaires, participants were invited to take part in a study on “Emotion in peoples’ daily encounters.” None declined to participate in the diary part and there was no monetary incentive. The University of Crete Psychology department research ethics committee approved the study.

### Procedure

Participants completed the individual differences (questionnaires) section prior to the event sampling data collection part. The questionnaires were preceded by an informed consent form indicating that completion of the questionnaire constituted proof of participants’ informed consent to take part in the study. Following this task, participants were briefed on the event sampling (diary) procedure which lasted for a period of 7 days after which records were returned to the laboratory individually.

### Individual Differences Measures

Participants completed a series of individual difference measures that included the Singelis (1994) Self-Construal Scale, a measure of trait or chronic self-construal (independence α = 0.67, interdependence α = 0.70, *r* (165) = -0.04, *p* > 0.50). The revised version of the SCS (Kwan et al., 1997) was used that consists of two orthogonal dimensions that measure the strength of independent and interdependent self-construal. Each subscale contains 15 items and responses were made on a seven point Likert-type scale (1 = strongly disagree, 7 = strongly agree). The SCS distinguishes between independent and interdependent self-construal at the individual level (Singelis, 1994). In two outlying cases the outliers were replaced with the mean.

Participants further completed a measure of social anxiety, the *Brief Fear of Negative Evaluation Scale* (BFNE, Leary, 1983, α = 0.93) and the *Positive and Negative Affect Scale* (PA and NA, respectively, Watson et al., 1988) which was completed at the end of four different days. For the purpose of this study the four day PA and NA scores were collated, a reflection of trait-level affect (α = 0.87 for PA and α = 0.90 for NA)^2^.

### Event Sampling (Diary) Task

Participants were told that this part of the study was about emotion in everyday social interactions. Following Wheeler and Nezlek (1977), they were instructed to use the Rochester Interaction Record to describe for seven days every social interaction they had that lasted 10 min or longer. Each participant was provided with a small booklet containing 40 interaction records. An interaction was defined as any encounter in which participants attended to one another and adjusted their behavior in response to one another. They were asked to fill out the forms as soon as possible after an interaction. For each interaction, participants reported their relationship with the other person(s) on a 6-point scale reflecting ordinal scaled levels of intimacy ranging from acquaintance to family member. In total, participants described 2557 interactions with acquaintances (18.7%), friends (17.2%), good friends (14.9%), best friends (20.1%), partners (12.3%), and family members (16.8%), (*M* = 2.29, *SD* = 1.18 per day).

#### Own Emotions

Participants described their own positive (happy, enthusiastic, interested, elated, calm, relaxed, satisfied, secure) and negative (angry, stressed, nervous, sad, bored, tired, rejected, ashamed) emotions during each social interaction. Participants also evaluated the other person’s emotional states (as positive or negative), their general satisfaction with the interaction as well as the degree to which they attended to other person’s emotions. For this they used 7-point Likert scales, anchored with 1 – *not at all* and 7 – *very much.* If more than one person was present during the interaction, participants were instructed to report on the person they interacted with most. Using three-level HLM latent variable analyses of scale scores (see Nezlek, 2012) social interaction level reliability was calculated as 0.80 for positive and 0.67 for negative emotions.

## Results

Table 1 presents results from zero order correlations between the study’s main individual differences variables. Independent self-construal was negatively correlated with Social anxiety, *r*(165) = -0.31, *p* < 0.001 and interdependent self-construal was positively correlated to social anxiety, *r*(165) = 0.41, *p* < 0.001. The two self-construal dimensions were unrelated to positive or negative affect over four days with correlations close to zero.

### Self Construal, Social Interaction Level Affect, and Social Interaction Outcomes

The data conform to a nested data structure, comprising two levels: the person-level and the social interaction level which is nested within persons. Therefore, two-level random coefficient models were calculated (Bryk and Raudenbush, 1992) using the software HLM 6.1 in which social interactions were the level 1 units of analysis, and individuals were the level 2 units. We first calculated the means and the within-subject (level 1, the social interaction level) and between-subject (level 2, person level) variances of the social interaction level variables (see Table 1). Inspection of the means suggests that overall, and in line with previous research (e.g., Nezlek et al., 2008) social interactions were perceived as largely positive rather than negative and participants reported experiencing satisfactory interactions and attending to other persons’ emotions.

In order to assess the influence participants’ independent and interdependent self-construal had on their satisfaction with the social interaction and on attending to others’ emotions, we used the following model that tested positive and negative affect (PA and NA) in social encounters as predictors at the social interaction level. PA and NA were centered on each participant’s average degree of positive and negative affect, respectively. Given the correlations between independent and interdependent self-construal and social anxiety, the analyses were conducted controlling for social anxiety in a second step.

Level 1:

$$y_{ij}=\beta_{0j}+\beta_{1j}(\mathrm{PA})+\beta_{2j}(\mathrm{NA})+r_{ij}$$

Level 2:

$$\beta_{0j}=\gamma_{00}+\gamma_{01}(\mathrm{Gender})+\gamma_{02}(\mathrm{Trait}\;\mathrm{PA})+\gamma_{03}(\mathrm{Independence})+\gamma_{04}(\mathrm{Interdependence})+\gamma_{05}(\mathrm{Social}\;\mathrm{Anxiety})+u_{0j}$$

$$\beta_{1j}=\gamma_{10}+\gamma_{11}(\mathrm{Gender})+\gamma_{12}(\mathrm{Trait}\;\mathrm{PA})+\gamma_{13}(\mathrm{Independence})+\gamma_{14}(\mathrm{Interdependence})+\gamma_{15}(\mathrm{Social}\;\mathrm{Anxiety})+u_{1j}$$

$$\beta_{2j}=\gamma_{20}+\gamma_{21}(\mathrm{Gender})+\gamma_{22}(\mathrm{Trait}\;\mathrm{PA})+\gamma_{23}(\mathrm{Independence})+\gamma_{24}(\mathrm{Interdependence})+\gamma_{25}(\mathrm{Social}\;\mathrm{Anxiety})+u_{2j}$$

The results are presented in Table 2 in two steps. The second step depicts the additional effect of Social anxiety in the relationships between independent and interdependent self-construal, positive emotion in social encounters, and social interaction outcomes. The upper panel presents what can be described as main effects. Generally, independent and interdependent self-construal were not directly related to social interaction outcomes. However, several significant effects were observed mainly as a function of positive affect at the social interaction level and interdependent self-construal further moderated those associations.

**Table 2:** Multilevel summary statistics.

	Mean	ICC1	Variance	
			
		% of variance within persons	Between-persons	Within-Persons
Satisfaction with the interaction	5.11	0.71	0.58	1.42
Attention to others’ emotion	5.29	0.62	0.74	1.26
Own positive emotions	4.51	0.63	0.56	0.92
Own negative emotions	1.99	0.57	0.56	0.77
Other positive emotions	5.39	0.71	0.52	1.29


**FIGURE 1:**
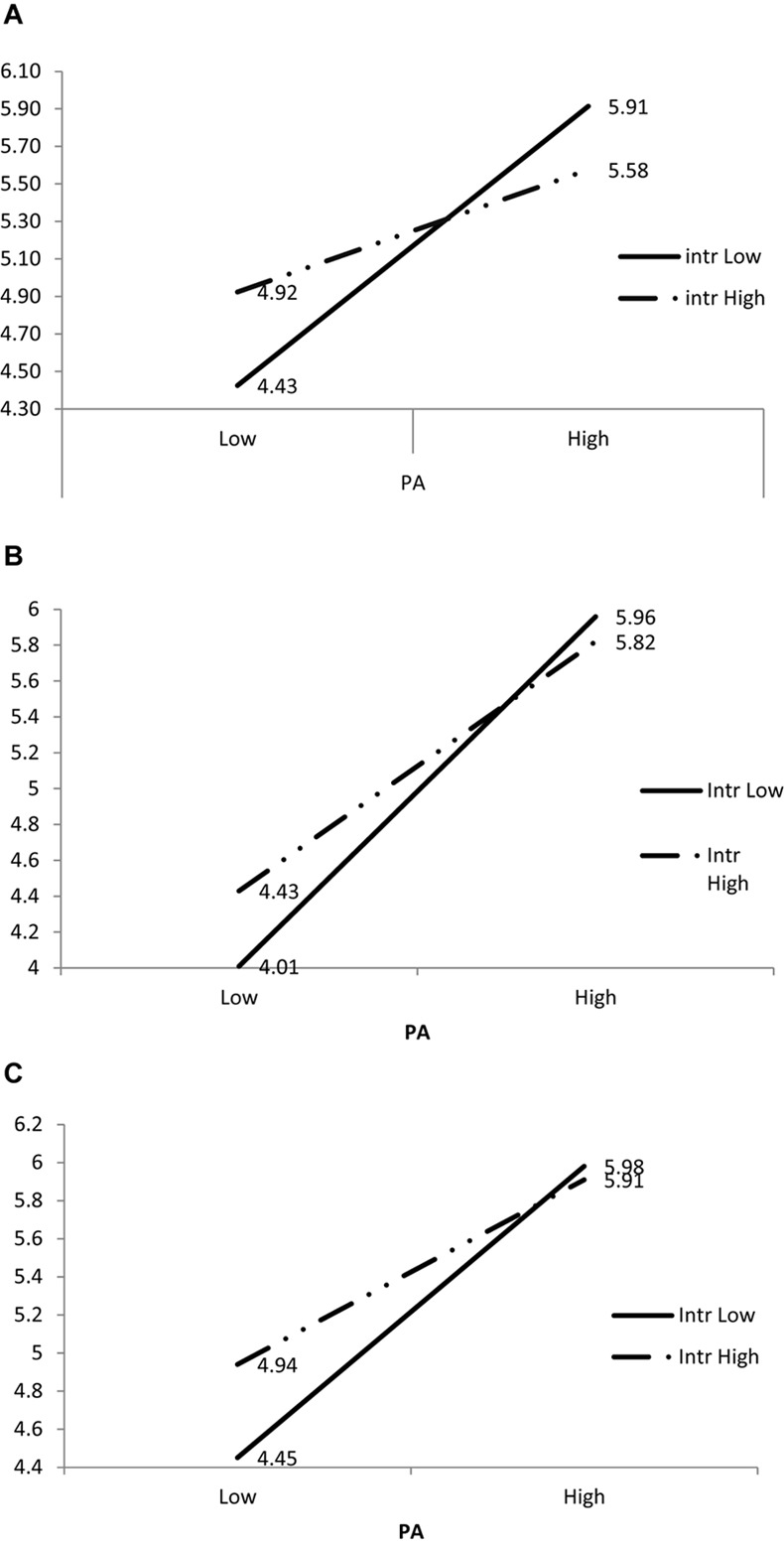
Plots depicting interaction between interdependent self-construal, positive and negative affect in social interaction and the three social interaction outcomes. **(A)** Effects of High and Low levels of Interdependent self-construal and PA on attending to others’ emotions. **(B)** Effects of High and Low levels of Interdependent self-construal and PA on satisfaction with the social interaction. **(C)** Effects of High and Low levels of Interdependent self-construal and PA on perceiving the other expressing positive emotion.

As expected, positive affect slope was moderated by interdependent self-construal. As depicted in Table 3, interdependence was a significant moderator of the positive emotion slope for: attending to others (γ_12_ = -0.22, *t* = -3.24, *p* = 0.002), satisfaction (γ_12_ = -0.16, *t* = -3.28, *p* = 0.002), and others expressing positive emotion (γ_12_ = -0.15, *t* = -2.60, *p* = 0.01). The moderating relationships of interdependent self-construal were consistent for all three social interaction outcomes. Positive emotion was positively related to the three social interaction outcomes and interdependent self-construal had a negative moderating coefficient suggesting that for those higher on interdependent self-construal the positive emotion slopes were less positive. To further probe these interactions I calculated predicted values for persons higher and lower in self-construal and higher and lower positive emotion slopes (Table 4). As can be seen in Figure 1A–C that graphically depict these interactions at low levels of positive emotion, higher interdependence was associated with higher social interaction quality compared to lower interdependence. At higher levels of positive emotion, however, higher interdependence was associated with lower attention to other and lower satisfaction compared to lower interdependent persons. The reported results were not meaningfully different when trait-level PA and NA were controlled. Levels of intimacy (acquaintance to family member) as a level 1 predictor were also controlled but did not return any significant findings or an interaction with individual level self-construal.

**Table 3:** Trait level self-construal and social anxiety as moderators of slopes between interaction outcomes and positive and negative affect.

	Attend To Other		Satisfied		Other expresses positive emotion	
			
	Step1	Step2	Step1	Step2	Step1	Step2
Intercept γ_*00*_	5.21	5.22	5.06	5.06	5.32	5.32
	(0.08)	(0.07)	(0.07)	(0.06)	(0.06)	(0.07)
Gender γ_*01*_	0.16^*^	0.16^*^	0.10	0.10	0.14^*^	0.14^*^
	(0.08)	(0.08)	(0.07)	(0.07)	(0.06)	(0.06)
Trait PA γ_*02*_	0.12^*^	0.11^*^	0.06	0.06	0.07	0.07
	(0.05)	(0.05)	(0.05)	(0.05)	(0.05)	(0.05)
Independent γ_*03*_	0.14	0.22^∧^	0.02	0.05	0.10	0.11
	(0.11)	(0.11)	(0.10)	(0.12)	(0.10)	(0.11)
Interdependent γ_*04*_	0.12	0.04	0.10	0.07	0.12^∧^	0.11
	(0.09)	(0.11)	(0.08)	(0.08)	(0.08)	(0.09)
Social Anxiety γ_*05*_		0.15^∧^		0.06		0.02
		(0.08)		(0.07)		(0.06)
**Positive Affect Slope**						
Intercept γ_*10*_	0.58^***^	0.57^***^	0.87^***^	0.87^***^	0.68^***^	0.67^***^
	(0.05)	(0.04)	(0.05)	(0.04)	(0.05)	(0.05)
Gender γ_*11*_	-0.01	-0.01	0.05	0.06	-0.01	-0.01
	(0.05)	(0.05)	(0.05)	(0.04)	(0.05)	(0.04)
Trait PA γ_*12*_	-0.05^*^	-0.06	-0.02	-0.04	0.02	0.01
	(0.05)	(0.05)	(0.04)	(0.04)	(0.04)	(0.04)
Independent γ_*11*_	0.06	0.13^∧^	0.05	0.11^∧^	0.03	0.09
	(0.07)	(0.08)	(0.06)	(0.06)	(0.07)	(0.07)
Interdependent γ_*12*_	-0.15^*^	-0.22^**^	-0.10^*^	-0.16^**^	-0.09	-0.15^*^
	(0.06)	(0.07)	(0.05)	(0.05)	(0.06)	(0.06)
Social Anxiety γ_*13*_		0.12^*^		0.11^*^		0.11
		(0.05)		(0.04)		(0.05)
**Negative Affect Slope**						
Intercept γ_*20*_	0.17^**^	0.17^**^	-0.44^***^	-0.44^***^	-0.30^***^	-0.30^***^
	(0.06)	(0.06)	(0.06)	(0.06)	(0.05)	(0.05)
Gender γ_*21*_	-0.05	-0.05	0.02	0.02	-0.01	-0.01
	(0.06)	(0.06)	(0.06)	(0.06)	(0.06)	(0.07)
Trait PA γ_*22*_	-0.05	0.01	-0.04	-0.04	-0.03	-0.04
	(0.05)	(0.06)	(0.05)	(0.05)	(0.06)	(0.05)
Independent γ_*21*_	0.10	0.11	-0.07	-0.01	-0.05	0.01
	(0.11)	(0.12)	(0.10)	(0.10)	(0.10)	(0.10)
Interdependent γ_*22*_	0.16^*^	0.15^∧^	-0.10	-0.15^*^	-0.03	-0.09
	(0.07)	(0.08)	(0.07)	(0.07)	(0.07)	(0.08)
Social Anxiety γ_*23*_		0.01		0.10^*^		0.11^∧^
		(0.06)		(0.05)		(0.07)


*Standard Error in parentheses. Positive and Negative affect slope denote the association between interaction-level PA and NA across all types of interactions. ^∗^*p* < 0.05, ^∗∗^*p* < 0.01, ^∗∗∗^*p* < 0.001, ^∧^*p* < 0.10.*

**Table 4:** Predicted values in social interaction outcomes for a combination of low and high Positive affect and Interdependent self-construal.

		Attend		Satisfied		Other positive	
				
		Low PA	High PA	Low PA	High PA	Low PA	High PA
Interdependence	Low	4.43	5.92	4.01	5.96	4.45	5.98
	High	4.92	5.58	4.43	5.82	4.94	5.91


Negative affect was associated with higher attention to the other but lower satisfaction and perceiving other as positive, and interdependence moderated the negative afffect attention to other association. Specifically, higher interdependence was associated with more attention to others in social interactions with more negative affect (albeit at non-significant level, γ_24_ = 0.14, *t* = 1.78, *p* = 0.075), yet with lower satisfaction of the interaction (γ_24_ = -0.15, *t* = -2.15, *p* = 0.033). Although partly supportive of H2, these results suggest that negative affective valence may differentially interpolate with interdependent self-construal.

When controlling for levels of intimacy, in addition to the above observed effects (which remained meaningfully the same), there was some indication that higher independent self-construal resulted in higher quality social interaction (see H2, higher attention to others: γ_13_ = 0.15, *t* = 1.86, *p* = 0.056, higher satisfaction, γ_13_ = 0.12, *t* = 1.94, *p* = 0.06, and higher perception of others’ emotions as positive γ_13_ = 0.11, *t* = 1.76, *p* = 0.06).

Finally, as can be observed in the second step of the multilevel regressions presented in Table 3, socially anxious persons seemed to profit from social interactions with higher positive affect. This is an interesting finding adding information on the relational outcomes of social anxiety in positive social interactions (e.g., Brown et al., 2007), which, however, goes beyond the scope of the current research.

## Discussion

Positive emotion has typically been considered a psychological resource with universal application, especially within people’s personal and social relationships (e.g., Lambert et al., 2013; Rohrer et al., 2018). However, depending on a person to social context fit, positive emotion may equally beneficial to everyone (Greenaway and Kalokerinos, 2017). Cultural norms and scripts can particularly determine how positive emotion is effectively regulated (Miyamoto and Ma, 2011). The present research brings to forth some of the first evidence, to our knowledge, that the relationship between positive emotion and social interaction quality can depend on a person’s chronic self-construal (higher interdependence) and the culture’s overarching cultural norms (collectivism).

During a week-long event sampling (diary) study, for participants higher in chronic interdependent self-construal positive emotion had a different association with perceptions of their social interaction quality, compared to persons who reported lower interdependent self-construal. At low levels of positive emotion, persons high on interdependent self-construal had higher social interaction quality than persons low on interdependent self-construal. At high levels of positive emotion, however, highly interdependent persons reported lower social interaction quality (lower satisfaction and a lower tendency to attend to the other person) than persons low in interdependence. Importantly, these findings had specifically to do with how emotion was experienced within social interactions, since positive and negative emotion were unrelated to interdependent or independent self-construal and controlling for trait positive and negative affect did not alter results.

Taken together, the above results suggest that for persons higher in chronic interdependent self-construal, lower levels of positive emotion are considered beneficial but higher levels can entail relational costs. A likely explanation for this pattern of results rests on that positive emotion in social encounters is typically down-regulated in cultures higher in collectivism in order to conserve harmony between people and within groups (Matsumoto, 1990). In collectivistic cultures and for persons higher in interdependent self-construal down-regulating and suppressing emotion is associated with some positive relational outcomes in personal (Le and Impett, 2013), social (Kafetsios et al., 2018), and work relationships (Kafetsios and Gruda, 2018). Hence, interactions that conform to this central cultural script are experienced as satisfactory for persons who are higher (as opposed to lower) in interdependent self-construal. Interactions that do not conform to this norm are not experienced as satisfactory. The results from the present study also suggest that scripts for the regulation of positive emotion in particular (e.g., Miyamoto and Ma, 2011) can extend to a variety of interactions with social acquaintances, friends, partners, and family. Positive emotion involves relational tendencies and social approach goals (Forgas, 2001; Fischer and Manstead, 2008) and is more strongly related to social interaction quality (Berry and Hansen, 1996; Campos et al., 2015).

Regarding the moderating role of self-construal in the association between negative emotion and social interaction quality, the results were mixed. In support of the person to culture fit hypothesis was evidence that higher interdependence strengthened the positive relationship between negative emotion and attending to the other person. Albeit supportive of the expected general pattern of cultural fit, this finding was different from previous research where interdependence was found to weaken the negative association between negative emotion and social interaction quality (Kafetsios et al., 2018). Moreover, there was also contradictory evidence that higher interdependence strengthened the negative association between negative emotion and satisfaction in social interaction. It is likely that the more detailed way that emotion was assessed in the present study (several different positive and negative emotions experienced), and the introduction of individual-level predictors such as social anxiety, may have played a role in the found associations. Surely, these findings highlight the differing functions of positive and negative emotion in social interaction (Berry and Hansen, 1996; Fischer and Manstead, 2008).

Overall, the present research provides a more nuanced picture of emotional fit within culture. A stream of research recently has highlighted emotions as predictive of acculturation levels to the host culture. Emotional fit within culture was particularly predictive of *relational* well-being (De Leersnyder et al., 2014). The present study involved persons from the same culture and their social encounters, the structuring situations that may underlie individuals’ emotional acculturation. Future research could examine how persons from different cultures and with different cultural self-construal may experience social interactions and how these may shape well-being in individualistic and collectivistic cultures (but also see Choi et al., 2018).

The results also tend to support a culturally situated social cognition perspective (Oyserman, 2011): emotion in dyadic encounters may prime culture-related scripts, especially in the case of chronic interdependence, a cultural orientation that is geared toward relating rules and obligations toward in-group members. Further indicative but inconclusive evidence for the moderating function positive emotion in social encounters can have was evidence that positive emotion was associated with higher quality social interactions for persons higher (as opposed to lower) independent self-construal. However, those effects did not all reach significance. These results beg following up with further experimental research that would test how positive emotional situations may differently prime independence and interdependence relation outcomes.

These results illustrate, in a naturalistic setting, how relational outcomes of independence and interdependence can depend on the specific structuring situations that give rise to individualistic or collectivistic mind sets (Hong et al., 2000; Oyserman and Sorensen, 2009). The results as a whole point to person to culture fit around a person’s self-construal and a key facet of social interaction, positive emotion. Interpersonal interactions are important for emotion construction (Fischer and Van Kleef, 2010), for well-being and for cultural understanding of the self. Hence, a ’fit’ between the central cultural mandate (collectivism), individual self-construal, and positive emotion in social interactions may modulate possible acculturation processes revolving around different emotions. At a theoretical level, the results suggest that any analysis of the role of emotion in social relationships and relational well-being has to take into account the immediate social situations within which emotions are experienced and expressed (Reis, 2008).

## Limitations and Future Directions

The results from this paper should be evaluated with a number of limitations in mind. Firstly, findings are based on self-report data and various factors can influence how people introspect on their own psychological processes. Secondly, results mainly concern a younger generation. Generally, younger generations are set to value autonomy and self-expression more than older generations and it is a question that requires further examination as to how the rules of expressing and experiencing positive emotion in social interaction extend to older generations. Thirdly, the list of emotions examined included, mainly, individual affective states. Future research could include assessment of a larger array of discrete emotions especially those that form the basis of social appraisals to engage or disengage from others (Kitayama et al., 2000).

Despite those limitations, the study has the added value that it examined people’s naturally occurring social interactions hence alleviating any doubts regarding likely demand characteristics presented in experimental settings. An additional advantage is that it took place in an interdependent culture outside of Asia. Much of the research on the topic adopted a typical East-West comparison in outcomes of interdependent and independent self-construal within cultures, ample and meaningful variation of those two main cultural orientations (Oyserman, 2011), and their effects can be observed as a result of individual variations in levels of independent and interdependent self-construal.

## Conclusion

Positive emotion has typically been considered an interpersonal resource (Gable et al., 2004) with universal effect. The results from the present study, however, suggest that cultural constructions of the self (interdependence) within an interdependent culture render can moderate the benefits of positive emotion depending of the level of positive emotion. Therefore, cultural self-construal and the related cultural scripts are important indicators of emotional fit within culture.

## Author’s Note

A previous version of this manuscript was presented at the 2018 Consortium for Research on Emotion Conference, University of Glasgow, under the title: Positive emotion in social encounters can lead to negative social interactions: Interdependent self-construal as a moderator.

## Ethics Statement

The University of Crete Psychology department research ethics committee approved the study. The questionnaires were preceded by an informed consent form indicating that completion of the questionnaire constituted proof of participants’ informed consent to take part in the study. Following this task, they were briefed on the event sampling (diary) procedure which lasted for a period of 7 days after which records were returned it to the laboratory individually.

It is stated:

“The University of Crete Psychology department research ethics committee approved the study. The questionnaires were preceded by an informed consent form indicating that completion of the questionnaire constituted proof of participants’ informed consent to take part in the study.”

## Author Contributions

The author confirms being the sole contributor of this work and has approved it for publication.

## Conflict of Interest Statement

The author declares that the research was conducted in the absence of any commercial or financial relationships that could be construed as a potential conflict of interest.

## Footnotes

1 Social norms are considered “The implicit or explicit rules a group has for the acceptable behaviors, values, and beliefs of its members” (Aronson et al., 2018, p. 505) whereas a script is defined as a “predetermined, stereotyped sequence of actions that defines a well-known situation” (Schank and Abelson, 1977, p. 41).

2 The study also measured emotional regulation, adult attachment orientation, ambivalence over emotion expression, Big Five Personality scale, group entitativity, and the Wong and Law emotional intelligence scale.
